# Dyshidrosiform Bullous Pemphigoid: Case Reports and Review

**DOI:** 10.7759/cureus.6630

**Published:** 2020-01-11

**Authors:** Philip R Cohen

**Affiliations:** 1 Dermatology, San Diego Family Dermatology, San Diego, USA

**Keywords:** blister, bullous, corticosteroid, dyshidrosiform, dyshidrosis, dyshidrotic, elderly, pemphigoid, pompholyx, vesicle

## Abstract

Bullous pemphigoid is an autoimmune blistering disorder that typically presents in elderly patients as pruritic tense subepidermal blisters on the lower trunk, axilla, and groin. It is caused by circulating and tissue-bound autoantibodies directed against bullous pemphigoid antigen 1 or bullous pemphigoid antigen 2 or both. Dyshidrosiform bullous pemphigoid is a rare variant of bullous pemphigoid, and it usually presents as itchy, potentially hemorrhagic, or purpuric blisters on the palms and/or soles of elderly individuals; subsequently, typical bullous lesions of bullous pemphigoid appear on other body sites. In our study, we report the features of two men with dyshidrosiform bullous pemphigoid and review the characteristics of individuals with this rare subtype of bullous pemphigoid. Including the men whose condition is described in this paper, at least 72 patients with dyshidrosiform bullous pemphigoid have been reported so far. However, complete features of the condition have not been described for all of the individuals.

Based on the cases reported so far, the condition was slightly more common in women and the onset of the disease, for most of the patients, occurred between the ages of 61 and 94 years. The patients usually presented with blisters on both their palms and soles (66%) or just their soles (31%); 77% of the patients had progression of bullous pemphigoid to other areas of their body. Whether hemorrhagic blisters or purpuric lesions are associated with dyshidrosiform bullous pemphigoid remains to be determined; these features were present in 91% of the 22 patients who were described in the case reports yet were only observed in 5% of the individuals from a single larger series of 20 patients. The mainstay of therapy for dyshidrosiform bullous pemphigoid is systemic corticosteroids, with or without topical corticosteroids, and/or systemic dapsone or immunosuppressants; nearly all of the patients showed improvement after the treatment was initiated. Similar to individuals with bullous pemphigoid, at least nine of the dyshidrosiform bullous pemphigoid patients, including both patients in this report, had either a neurologic condition (seven patients) or both a neurologic condition and a psychiatric disorder (two patients). Usually, an autoimmune bullous disease, particularly dyshidrosiform bullous pemphigoid, is not initially considered in patients who present with blisters restricted to the palms and/or soles. Indeed, the lesion morphology of dyshidrosiform bullous pemphigoid mimics several other conditions that are characterized by blisters on the hands and feet, such as allergic and irritant contact dermatitis, chronic bullous disease of childhood, cutaneous T-cell lymphoma, dermatophyte infection, dyshidrosis or pompholyx, epidermolysis bullosa acquisita, erythema multiforme, herpes gestationis, lichen planus, linear IgA disease, scabies, and systemic contact dermatitis. In conclusion, the possibility of dyshidrosiform bullous pemphigoid should be considered in elderly individuals who present with the new onset of palmar and/or plantar blisters that are either recurrent or recalcitrant to therapy or would subsequently also appear on other areas of the body.

## Introduction

Bullous pemphigoid is an autoimmune blistering condition that usually occurs in elderly individuals. The immunopathogenesis of the disease is attributed to circulating and tissue-bound autoantibodies directed against bullous pemphigoid antigen 230 (bullous pemphigoid antigen 1) or bullous pemphigoid antigen 180 (bullous pemphigoid antigen 2) or both [1-3]. The condition typically presents as pruritic tense subepidermal blisters. In addition to the lower trunk, the lesions frequently appear on the proximal flexural aspects of the arms (near the axilla) and legs (near the groin). The lesions can be localized or widespread [1-3].

Unusual clinical variants of bullous pemphigoid have been observed in literature [1-3]. Dyshidrosiform bullous pemphigoid refers to the condition when the blisters are initially or persistently localized to the palms and soles [4-19]. In our study, we report the features of two men with dyshidrosiform bullous pemphigoid and review the characteristics of individuals with this rare subtype of bullous pemphigoid.

## Case presentation

Case 1

A 61-year-old Filipino man presented with a two-month history of tender blisters on his feet. The lesions had made it difficult for him to walk. A week before his evaluation, he had also begun to develop red itchy lesions on his upper legs and central upper back. His past medical history was only significant for hypertension for which he was receiving atenolol daily.

**Figure 1:**
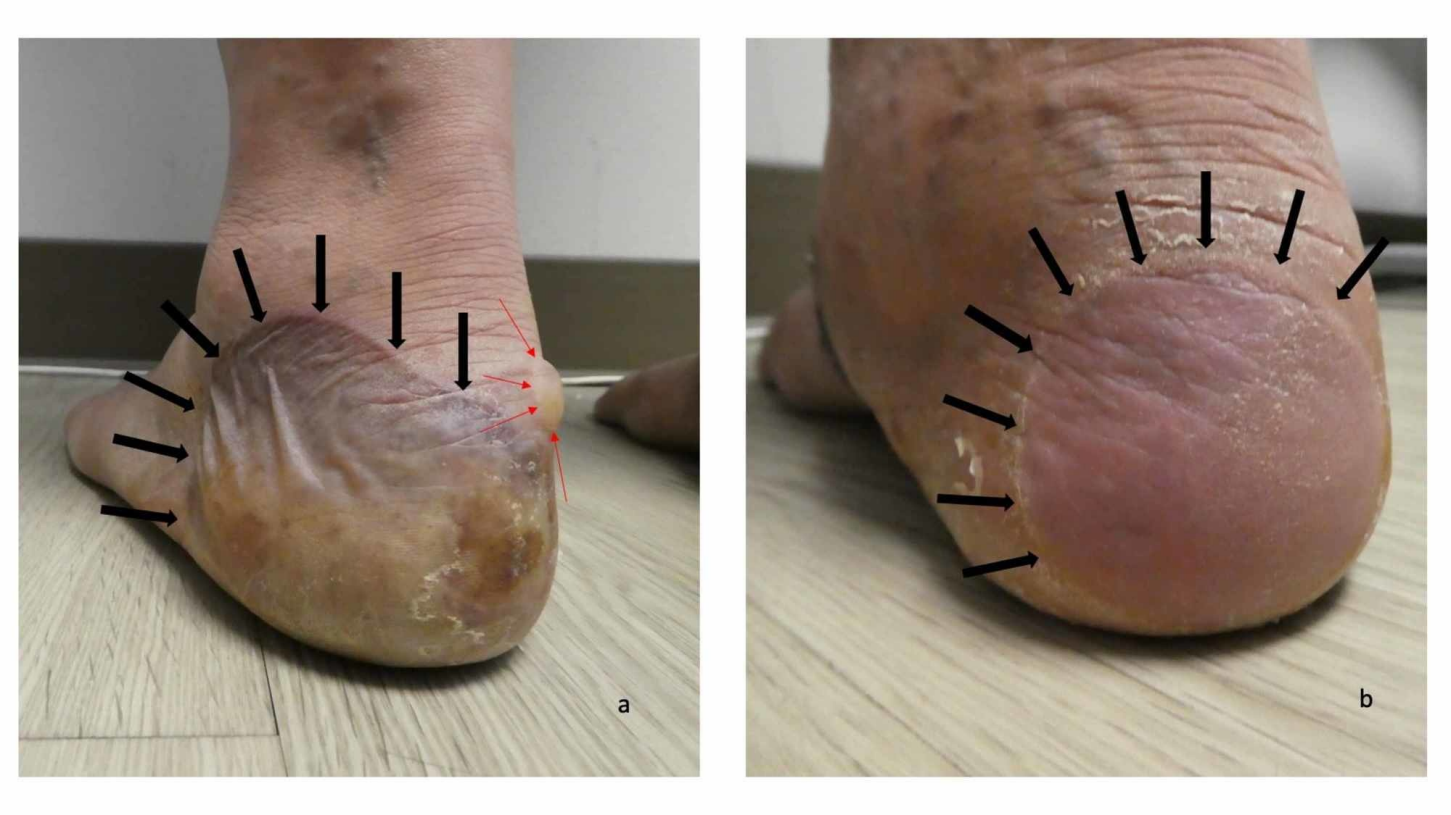
Dyshidrosiform bullous pemphigoid presenting as plantar blisters in a 61-year-old man The posterior and plantar left heel (a) and right heel (b) show a tender large flaccid hemorrhagic blister (a) and a flattened blister (b) as the initial clinical manifestations of dyshidrosiform bullous pemphigoid; each of these blisters is outlined by black arrows. A tense blister containing clear fluid (red arrows) is present on the medial area of his left heel (a)

**Figure 2:**
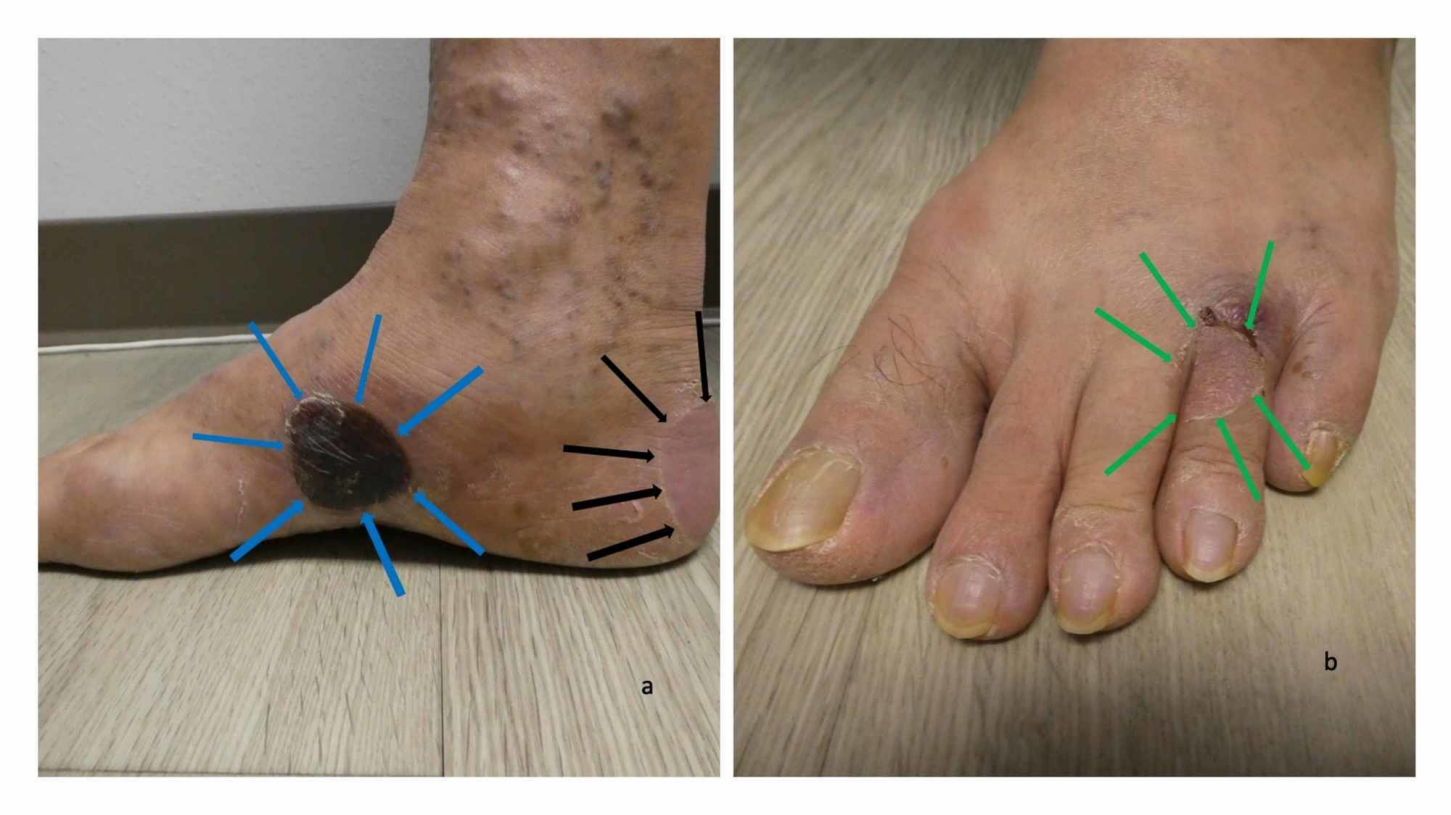
Blisters from dyshidrosiform bullous pemphigoid on the feet of a 61-year-old man The medial view of the right foot (a) shows a black-roofed and flattened blister on the instep (blue arrows) and another flattened blister that is primarily on the posterior heel and extends to the plantar foot (black arrows). The left dorsal foot (b) has a deroofed blister on the proximal fourth toe (green arrows)

**Figure 3:**
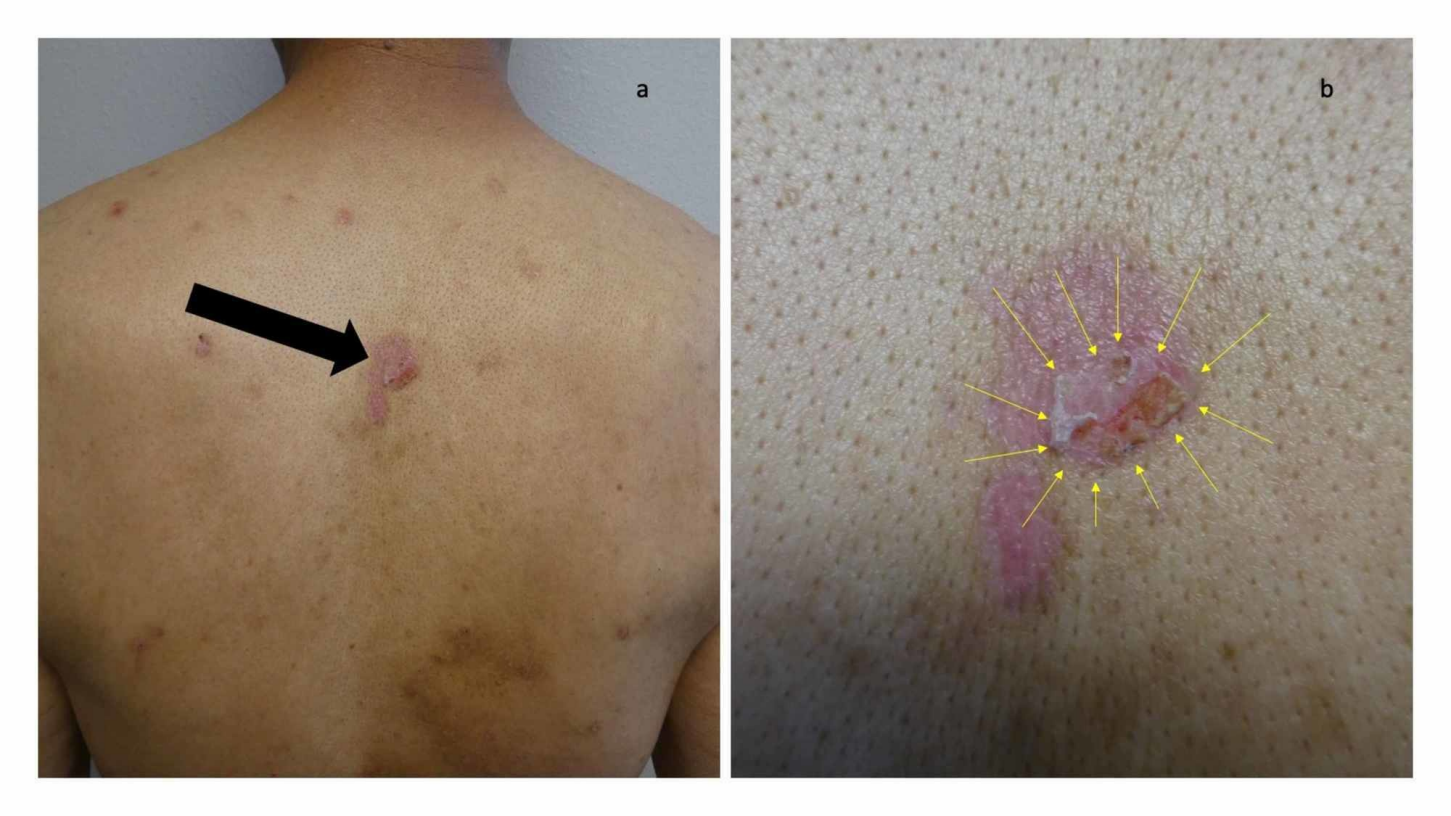
Erythematous urticarial dermal plaques and blisters on the back of a man with dyshidrosiform bullous pemphigoid Distant (a) and closer (b) views show several erythematous dermal plaques. A larger red urticaria-appearing plaque on the central upper back (black arrow), consistent with the urticarial stage of bullous pemphigoid, is present. In the center of the plaque, a blister (yellow arrows) has ruptured

**Figure 4:**
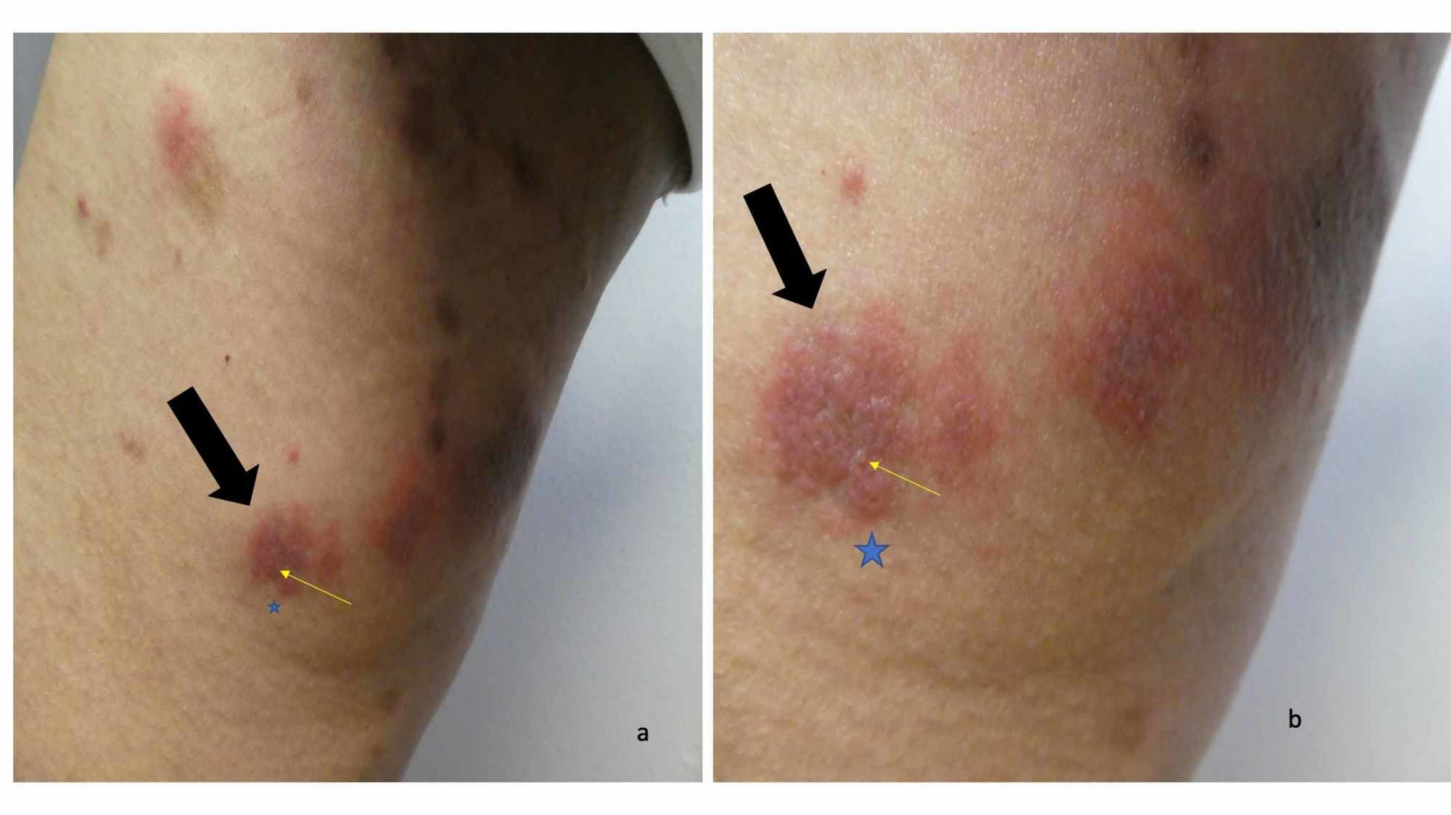
Erythematous urticarial dermal plaques with small papules on the right thigh of a man with dyshidrosiform bullous pemphigoid Distant (a) and closer (b) views of the right thigh show several red dermal plaques. A larger erythematous dermal plaque (black arrow) has a small papule (the tip of the yellow arrow); this was the location of the skin biopsy for hematoxylin and eosin staining which was consistent with the urticarial stage of bullous pemphigoid. The skin biopsy for direct immunofluorescence (blue star) was located adjacent to the skin lesion on the right thigh and demonstrated findings that were diagnostic for bullous pemphigoid

Cutaneous examination revealed painful, bilateral, large hemorrhagic blisters on posterior and plantar heels (Figure 1); there were no mucosal lesions. In addition, he had a flattened blister with a black roof on the instep of his right foot, and a blister whose roof had become detached was also present on the dorsal left foot on the proximal fourth toe (Figure 2). Erythematous urticarial dermal plaques were present on his central upper back (with a ruptured blister) (Figure 3) and the proximal medial thighs (with small papules) (Figure 4).

Skin biopsies of lesions on the right thigh and left ankle were performed for routine hematoxylin and eosin staining. Microscopic examination of the right-thigh biopsy showed scattered foci of spongiosis in the epidermis; in the papillary dermis, there was a band-like infiltrate of lymphocytes and numerous eosinophils, edema, and early subepidermal vesiculation. The left-ankle biopsy demonstrated a subepidermal blister with an inflammatory infiltrate consisting of lymphocytes, histiocytes, and eosinophils in the papillary dermis.

A second biopsy immediately adjacent to the right-thigh lesion was performed for direct immunofluorescence. Staining for immunoglobulin G (IgG) and C3 showed a smooth linear band of immunoreactant deposition at the dermoepidermal junction. Staining for immunoglobulin A (IgA), immunoglobulin M (IgM), and fibrinogen was negative. The hematoxylin and eosin-stained skin biopsy from the right thigh were consistent with the urticarial stage of bullous pemphigoid. Both the left- ankle (hematoxylin and eosin) and the right-thigh (direct immunofluorescence) biopsies were diagnostic for bullous pemphigoid. The correlation of the clinical history and histopathology and immunopathology established the diagnosis of dyshidrosiform bullous pemphigoid.

Serologic laboratory studies were also performed. Quantitative indirect immunofluorescence to anti-skin autoantibodies demonstrated a basement membrane zone (BMZ) staining pattern at a high titer of 1:160, confirming the diagnosis of bullous pemphigoid. The patient's eosinophil count was normal; however, his serum immunoglobulin E (IgE) level was very elevated at 7,582 UI/ml (normal: <115 UI/ml). Treatment was initiated with oral prednisone (60 mg daily) and topical betamethasone dipropionate 0.05% cream (twice daily). Within one week the lesions on his heels had almost completely healed; after four weeks, there were no new blisters. He began to taper the prednisone by 10 mg every other week; however, he developed new blisters when the daily dosage decreased from 30 mg to 20 mg.

Additional workup revealed that his QuantiFERON-TB Gold (QFT-G) test was positive. His chest roentgenogram was normal and he had no respiratory or constitutional symptoms consistent with active tuberculosis; his latent tuberculosis infection was treated with rifampin 600 mg daily for four months. During his latent tuberculosis treatment, he was maintained on 30 mg of prednisone daily without any new blisters appearing. The patient completed his rifampin treatment. He was started on azathioprine (after confirming that his thiopurine methyltransferase enzyme activity was normal) as a corticosteroid-sparing agent, initially at 50 mg twice daily and subsequently at 100 mg in the morning and 50 mg in the evening. The daily prednisone dosage of 30 mg was changed to an alternate-day dosage of 60 mg and slowly tapered over the next six months.

There were no new blisters and he was able to stop the prednisone; he is currently being maintained on azathioprine. During the period of corticosteroid tapering, he developed Parkinsonism. However, his symptoms improved after starting treatment with carbidopa and levodopa.

Case 2

A 65-year-old Mexican man presented with a three-month history of a skin rash and recurrent blisters. He was a resident at a skilled nursing facility and had multiple medical problems including ankylosing spondylitis, chronic pain syndrome, cognitive impairment, dysphagia with a feeding gastrostomy tube, epilepsy since childhood with occasional seizures, manic depressive disorder, and weakness. His long-term seizure medications included carbamazepine, phenobarbital, and phenytoin; citalopram had been added three years ago for the management of his psychiatric disorder.

Initially, blisters appeared on both of his feet. Within a month, he developed a lesion on his arms and legs. His physicians initially suspected a scabies infestation and he was treated with permethrin 5% cream; however, his skin condition persisted. Subsequently, a skin infection was also considered and he received several courses of oral antibiotics without improvement.

**Figure 5:**
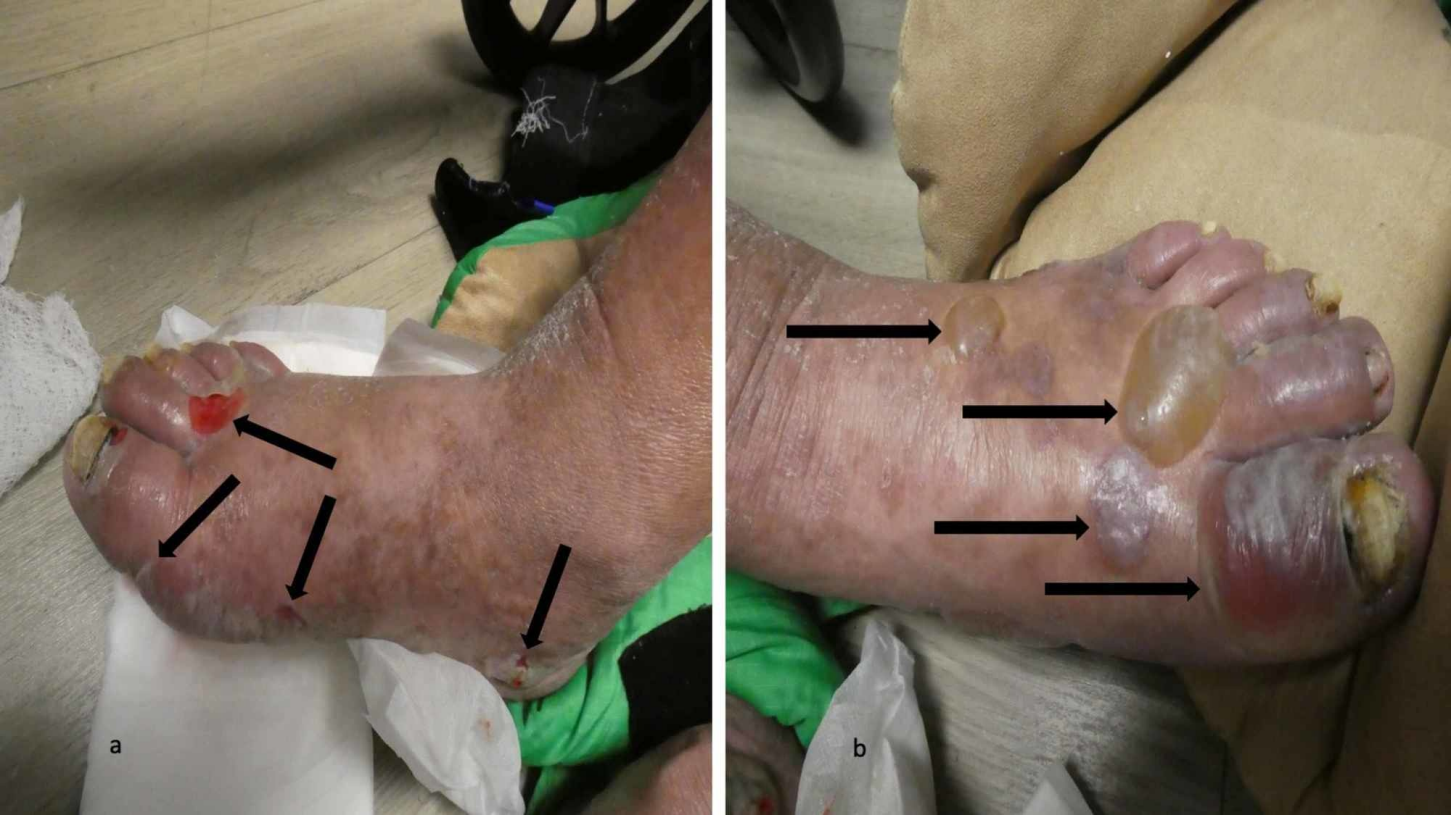
Dyshidrosiform bullous pemphigoid presenting as plantar blisters in a 65-year-old man The right foot (a) and left foot (b) show hemorrhagic and deroofed blisters (black arrows) on both the dorsal and lateral surface of his feet and toes; some of the blisters also extend to the soles of his feet

**Figure 6:**
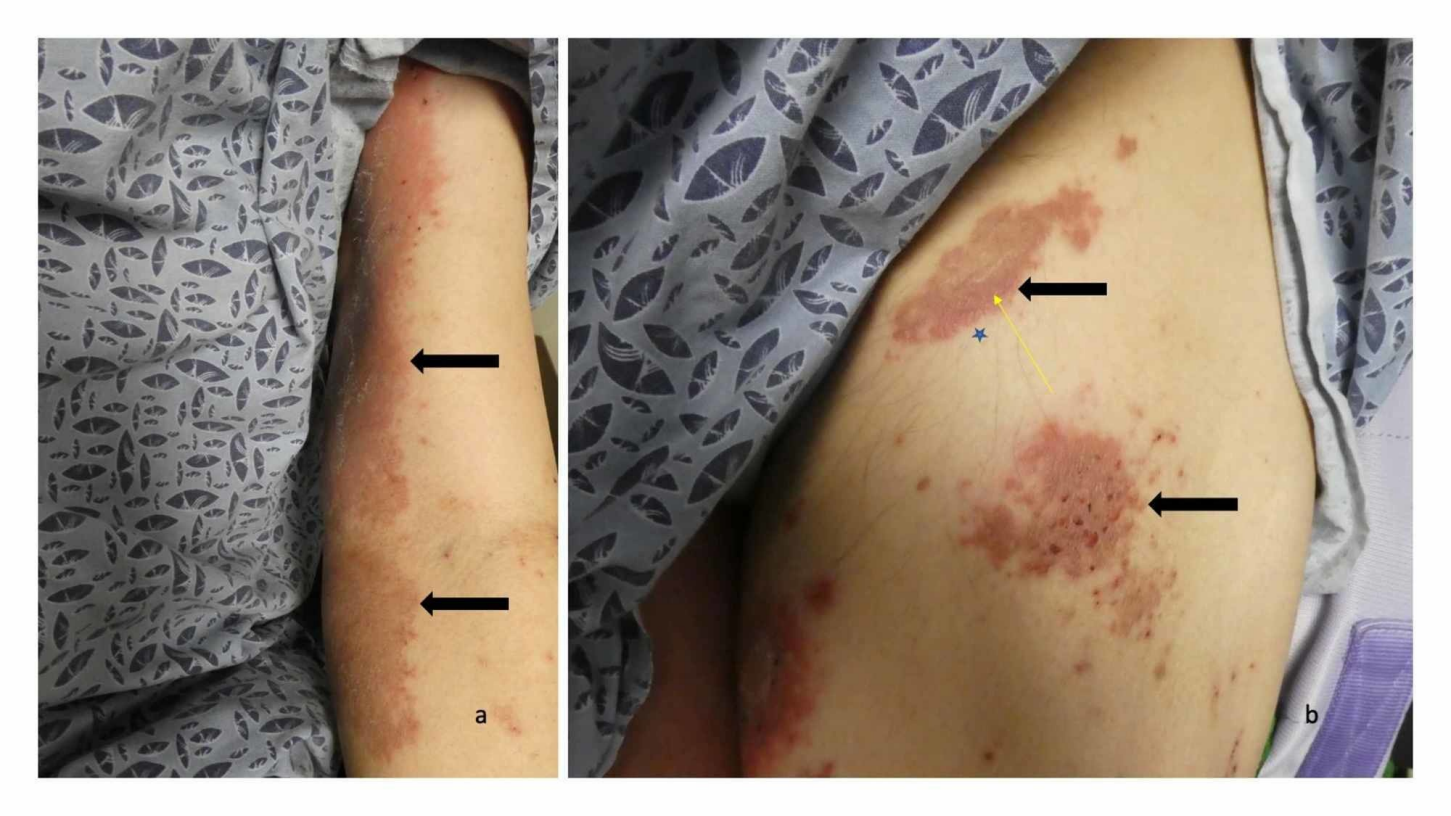
Erythematous urticarial dermal plaques on the left arm and left leg of a man with dyshidrosiform bullous pemphigoid The proximal left arm (a) and the proximal left thigh (b) show several erythematous dermal plaques (black arrows). The skin biopsy on his left thigh (b) for hematoxylin and eosin staining (the tip of the yellow arrow) show pathologic changes that are consistent with the urticarial stage of bullous pemphigoid. The skin biopsy for direct immunofluorescence (blue star) is located adjacent to the skin lesion on the left thigh and demonstrate findings that are consistent with bullous pemphigoid

Three months after the onset of his cutaneous condition, he was brought to see a dermatologist for an evaluation. Cutaneous examination showed hemorrhagic blisters on both the dorsal and plantar surfaces of his feet and toes (Figure 5). There were no mucosal lesions. Erythematous plaques were present on his arms and legs (Figure 6); biopsies from a left thigh plaque and adjacent skin were performed for routine staining and direct immunofluorescence, respectively. Initial management included soaks to feet with aluminum acetate solution and application of triamcinolone 0.1% cream to the lesions on his body; both interventions were to be done three times daily.

Microscopic examination of the hematoxylin and eosin-stained specimen showed eosinophilic spongiosis (showing eosinophils being present in the widened spaces between the epidermal keratinocytes), marked edema in the papillary dermis, and abundant eosinophils with some lymphocytes present in the inflammatory infiltrate in the upper dermis. These changes were consistent with those of the urticarial stage of bullous pemphigoid. The direct immunofluorescence stained specimen showed a linear band of both IgG and C3, with no staining for IgA, IgM, and fibrinogen, deposited at the dermoepidermal junction. These findings were diagnostic for bullous pemphigoid. Correlation of the clinical presentation of blisters on the feet and the pathology results (or routine-stained and immunofluorescence-stained specimens) established the diagnosis of dyshidrosiform bullous pemphigoid.

**Figure 7:**
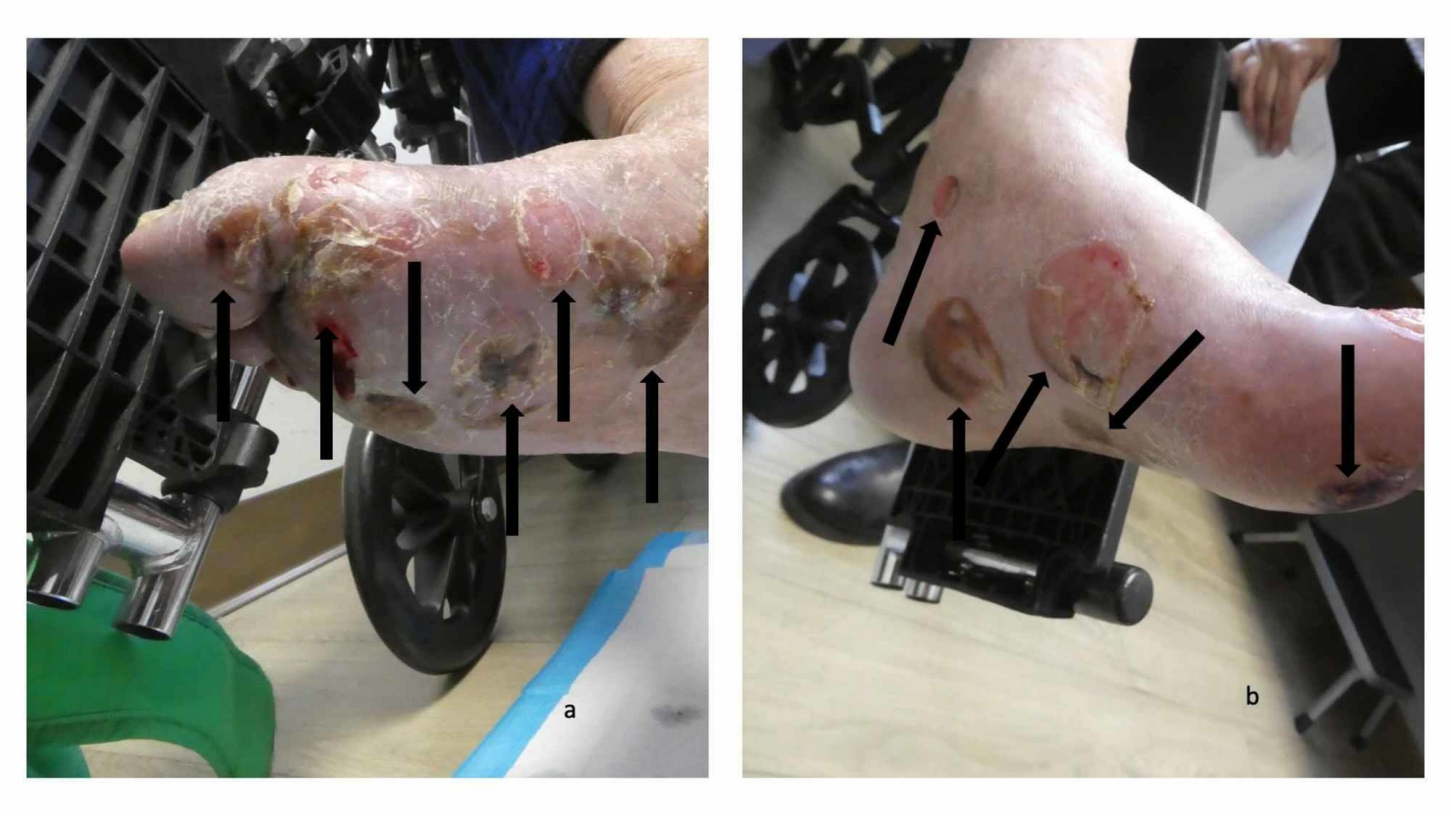
Blisters on the feet of a man with dyshidrosiform bullous pemphigoid Numerous flattened and deroofed blisters (black arrows) seen on the plantar and medial surfaces of the right foot (a) and left foot (b)

**Figure 8:**
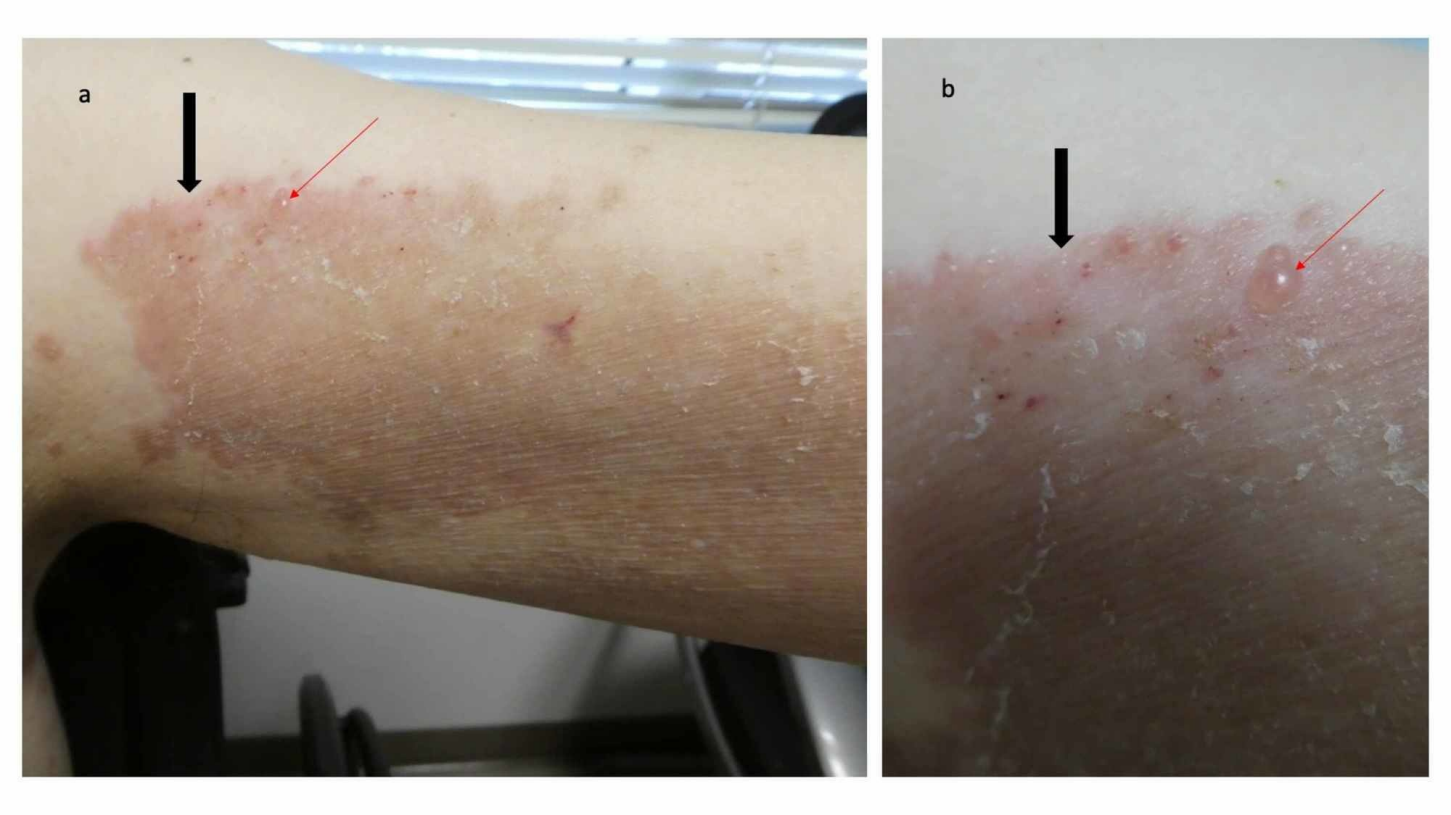
Erythematous plaque with vesicles on the left arm of a man with dyshidrosiform bullous pemphigoid The large confluent red dermal plaque on the proximal left arm (black arrow) has small vesicles; one of the vesicles is located at the tip of the red arrow

The patient returned after one week. Several of the blisters on his feet had flattened (Figure 7); however, small vesicles were noted on his arm plaques (Figure 8). His topical care was continued and he was started on 60 mg of prednisolone, which was to be administered through his gastrostomy tube each morning. Follow-up, one week later, showed significant improvement of both his feet and body; there were no new blisters and the erythematous plaques on his arms and legs were nearly resolved.

## Discussion

Dyshidrosiform bullous pemphigoid is a unique variant of bullous pemphigoid. The morphology of the lesions mimics vesicular hand or foot dermatitis. In fact, the clinical differential diagnosis not only includes dyshidrosis or pompholyx, but also allergic and irritant contact dermatitis, chronic bullous disease of childhood, cutaneous T-cell lymphoma (vesicular palmoplantar variant), dermatophyte infection (bullous), epidermolysis bullosa acquisita, erythema multiforme, herpes gestationis, lichen planus (bullous), linear IgA disease, scabies, and systemic contact dermatitis. The possibility of an autoimmune bullous disease is usually not entertained, particularly in individuals for whom the lesions are restricted to the palms or soles or both [4-19].

Dyshidrosiform bullous pemphigoid was initially described by Levine et al. in 1979 [4]. They described a 72-year-old man who initially presented with a vesicular eruption of the hands (that resolved in two weeks after treatment with tar soaks and topical corticosteroid application) and subsequently developed large tense bullae on his feet which cleared after two weeks of oral prednisone 40 mg daily. Blisters continued to periodically appear on his feet; in addition, one episode was also associated with truncal lesions and an erosion on his pharynx. Prior to skin biopsy confirmation of the diagnosis of bullous pemphigoid, he had also been treated with erythromycin, intramuscular corticosteroid, and griseofulvin. After establishing the diagnosis, he was eventually treated with dapsone 50 mg thrice daily for seven months; all of his lesions resolved after five days and there was no recurrence at follow-up four months after stopping the treatment [4].

Since the publication of Levine et al.’s paper, at least 71 additional patients, including the individuals in this report, with dyshidrosiform bullous pemphigoid have been reported in the literature. Most of the reports (24 papers) only described a single patient. However, there were two reports with two patients, four reports with three patients, one report with four patients, one report with nine patients, and one report with 20 patients (one of whom was previously described) [4-19]. Also, three larger studies of dyshidrosiform bullous pemphigoid have been performed [17-19]. One group of investigators only identified three patients with dyshidrosiform bullous pemphigoid in 86 individuals with bullous pemphigoid, who were treated in the largest teaching hospital in Taiwan from 1977 to 1994. The three patients predominantly developed vesicles and bullae on their palms and soles that were difficult to differentiate from pompholyx. All of the palm and sole lesions responded to treatment with prednisolone 30-40 mg per day [19].

However, the other two research groups observed 28% (20 of 71 patients) and 45% (nine of 20 patients) of individuals with palmar and/or plantar lesions in their series of bullous pemphigoid patients [17,18]. The palm and/or plantar lesions were the presenting feature in four of the 20 patients [18]. Also, only one of the 20 patients had hemorrhagic bullae [6,18].

The third study took place during a period of three years and included 20 bullous pemphigoid patients from Stockholm, Sweden; there were nine dyshidrosiform bullous pemphigoid patients, two of whom only had palm and/or sole lesions. The investigators emphasized that all nine of the dyshidrosiform bullous pemphigoid patients had prodromal symptoms, such as an eczematous eruption (three patients), a papular eruption (three patients), an eczematous and papular eruption (one patient), an intertriginous eruption (one patient), or an urticarial and papular eruption (one patient) for greater than (five patients) or less than (four patients) three months [17]. Based on the increased number of bullous pemphigoid patients with palm and/or sole lesions observed in the latter two studies, it is conjectured that dyshidrosiform bullous pemphigoid occurs more commonly than implied by the publication of patient descriptions in the individual case reports.

Based on the studies, epidemiology information was available for 38 of the 72 dyshidrosiform bullous pemphigoid patients. The onset age of dyshidrosiform bullous pemphigoid ranged from 20 years to 94 years (median: 76 years); however, only three of the patients were in their twenties. The age of the other 35 individuals ranged from 61 to 94 years [4-19]. Dyshidrosiform bullous pemphigoid was only slightly more common in women (20 patients) than men (18 patients). The onset age in women ranged from 23 years to 94 years (median: 77 years). Similarly, the onset age in men ranged from 20 years to 92 years (median: 75 years) [4-19]. Many of the patients initially had blisters on both their palms and soles (19 of 29 individuals, 66%) [4-19]. However, some of the patients only presented with lesions on either their palms (one of 29 individuals, 3%) or their soles (nine of 29 individuals, 31%).

Some of the researchers emphasized the association of hemorrhagic blisters or purpuric lesion with dyshidrosiform bullous pemphigoid [6,7]. The blisters were hemorrhagic and purpura was present in 91% of the patients (20 of 22 individuals) whose lesions were described in their individual case reports [4-16]. In contrast, only one of the 20 patients (5%) from a large series of patients had hemorrhagic lesions [18]. Similar to both of the patients described in this report, 77% of the patients (23 of 30 individuals) had progression of bullous pemphigoid to other areas of their body [4-19]; however, oral lesions were only described in three patients [6,11,16]. The duration of time between the onset of lesions on the palms and/or soles and new blisters on other body sites ranged from one week to seven months (median: seven weeks). In common with nine dyshidrosiform bullous pemphigoid patients who had prodromal symptoms prior to the onset of their blisters [17], one man also developed pruritic papules on his arms and upper back prior to the appearance of blisters on his palms [15].

All of the patients had histopathologic confirmation of their bullous pemphigoid diagnosis [4-19]. This not only included hematoxylin and eosin-stained sections of formalin-fixed lesional skin tissue specimens, but also immunofluorescence (direct, indirect or both) studies. Only a small number of patients had enzyme-linked immunosorbent assay or Western blot testing for autoantibodies against bullous pemphigoid antigen 1 or bullous pemphigoid antigen 2 or both [11,14]. Treatment of dyshidrosiform bullous pemphigoid was described for 32 of the patients [4-19]. Systemic corticosteroids-starting dosage ranging from 10-80 mg daily (median: 30 mg daily) was used in the management of most (26 individuals, 81%) of the patients. In fact, one group of investigators was able to successfully treat dyshidrosiform bullous pemphigoid patients with a lower daily dosage of prednisone [6].

Other interventional agents were also used in the treatment of dyshidrosiform bullous pemphigoid patients [4-19]. These included topical corticosteroids (12 individuals), dapsone (seven individuals whose dosage ranged from 50-200 mg daily; median: 150 mg daily), oral antibiotics such as erythromycin, doxycycline, or tetracycline (three individuals), and nicotinamide (one individual). Immunosuppressant agents were also used either alone or as a corticosteroid agent: azathioprine (three individuals whose dosage ranged from 100-150 mg daily; median: 100 mg daily) and cyclophosphamide (one individual whose daily dosage was 100 mg). Nearly all of the patients improved with the treatment [4-19]. Resolution of lesions occurred within one week to one month; however, recurrent episodes of palm and/or sole lesions were not uncommon either during tapering or after stopping of the systemic treatment. Indeed, many patients were still receiving therapy when they were reported. Two of the patients died from conditions deemed to be unrelated to bullous pemphigoid or its treatment, one from respiratory failure and the other from cardiac arrest [11,16].

An association between bullous pemphigoid and neurologic or psychiatric disorders has been identified [1-3]. Both of the men in this report had neurologic disorders, either epilepsy or Parkinsonism. In addition, at least seven of the other dyshidrosiform bullous pemphigoid patients had a neurologic disorder: cerebrovascular accidents (two men and one woman) [6,11,15], Parkinsonism (two women) [14,16], peripheral neuropathy (one woman) [16], and senile dementia (one woman) [11]. One of the men in this report also had manic depression syndrome. Similarly, one of the women patients suffered from depression [14]. Whether there is an increased incidence of neurologic and psychiatric conditions in dyshidrosiform bullous pemphigoid patients as compared to individuals with bullous pemphigoid without dyshidrosiform-like lesions remains to be determined.

## Conclusions

Dyshidrosiform bullous pemphigoid is a rarely described variant of bullous pemphigoid. Similar to idiopathic bullous pemphigoid, dyshidrosiform bullous pemphigoid typically presents with pruritic lesions in elderly individuals; the hemorrhagic or purpuric blisters on the palms and/or soles are often followed by the development of typical bullous lesions on other body sites. Nearly all dyshidrosiform bullous pemphigoid patients improve after the diagnosis is established and treatment is initiated. The mainstay of therapy is systemic corticosteroids, with or without topical corticosteroids, and/or systemic dapsone or immunosuppressants.

## References

[1] Bernard P, Antonicelli F (2017). Bullous pemphigoid: a review of its diagnosis, associations and treatment. Am J Clin Dermatol.

[2] Genovese G, Di Zenzo G, Cozzani E, Berti E, Cugno M, Marzano AV (2020). New insights into the pathogenesis of bullous pemphigoid: 2019 update. Front Immunol.

[3] Miyamoto D, Santi CG, Aoki V, Maruta CW (2019). Bullous pemphigoid. An Bras Dermatol.

[4] Levine N, Freilich A, Barland P (1979). Localized pemphigoid simulating dyshidrosiform dermatitis. Arch Dermatol.

[5] Liu HN, Su WP, Rogers RS 3rd (1986). Clinical variants of pemphigoid. Int J Dermatol.

[6] Barth JH, Fairris GM, Wojnarowska F, White JE (1986). Haemorrhagic pompholyx is a sign of bullous pemphigoid and an indication for low-dose prednisolone therapy. Clin Exp Dermatol.

[7] Duhra P, Ryatt KS (1988). Haemorrhagic pompholyx in bullous pemphigoid. Clin Exp Dermatol.

[8] Mohr C, Duschet P, Bonsmann G, Luger TA, Gschnait F, Schwarz T (1993). Dyshidrosiform bullous pemphigoid. (Article in German). Hautarzt.

[9] Beylot-Barry M, Doutre MS, Beylot C (1995). Dyshidrotic pemphigoid. (Article in French). Ann Dermatol Venereol.

[10] Braun B, Baima B, Sticherling M (2002). Bullous pemphigoid manifesting as dyshidrotic eczema and prurigo nodularis. (Article in German). Hautarzt.

[11] Sugimura C, Katsuura J, Moriue T, Matsuoka Y, Kubota Y (2003). Dyshidrosiform pemphigoid: report of a case. J Dermatol.

[12] Patrizi A, Rizzoli L, Benassi L, Neri I (2003). Another case of dyshidrosiform pemphigoid. J Eur Acad Dermatol Venereol.

[13] Kim YJ, Kim MY, Kim HO, Park YM (2004). Dyshidrosiform bullous pemphigoid. Acta Derm Venereol.

[14] Forschner A, Fierlbeck G (2005). Localized pemphigoid on the soles of both feet. Int J Dermatol.

[15] Seike M, Nakajima K, Ikeda M, Kodama H (2006). Coexistence of nodular and dyshidrosiform pemphigoid. J Dermatol.

[16] Basseri S, Ly TY, Hull PR (2018). Dyshidrotic bullous pemphigoid: case report and review of literature. J Cutan Med Surg.

[17] Asbrink E, Hovmark A (1981). Clinical variations in bullous pemphigoid with respect to early symptoms. Acta Derm Venereol.

[18] Barth JH, Venning VA, Wojnarowska F (1988). Palmo-plantar involvement in auto-immune blistering disorders—pemphigoid, linear IgA disease and herpes gestationis. Clin Exp Dermatol.

[19] Chang YT, Liu HN, Wong CK (1996). Bullous pemphigoid—a report of 86 cases from Taiwan. Clin Exp Dermatol.

